# Pregnant women’s clinical characteristics, intrapartum interventions, and duration of labour in urban China: a multi-center cross-sectional study

**DOI:** 10.1186/s12884-020-03072-x

**Published:** 2020-07-02

**Authors:** Chunyi Gu, Xiaojiao Wang, Zhijie Zhang, Simone Schwank, Chunxiang Zhu, Zheng Zhang, Xu Qian

**Affiliations:** 1 Department of Maternal, Child and Adolescent Health, School of Public Heath, Fudan University, Shanghai, China; 2 Department of Nursing, Obstetrics & Gynaecology Hospital of Fudan University, Shanghai, China; 3 Department of Epidemiology, School of Public Heath, Fudan University, Shanghai, China; 4 Department of Women and Children’s Health, Reproductive Health, Karolinska Institutet, Stockholm, Sweden; 5 Global Health Institute, Fudan University, Shanghai, China

**Keywords:** Childbirth, Intrapartum intervention, Labour duration, Normal labour

## Abstract

**Background:**

There is an increasing global trend towards the widespread over-medicalisation of labour and childbirth. The present study aimed to investigate pregnant women’s clinical characteristics, intrapartum interventions, duration of labour and its associated factors; and to compare the differences of these variables between nulliparas and multiparas in China.

**Methods:**

A multi-center cross-sectional study was carried out in three tertiary hospitals of Fudan University in Shanghai, China. A total of 1523 participants were approched and assessed for eligibility. Data on women’s sociodemographic characteristics, intrapartum interventions, and duration of labour were measured and collected. Kaplan-Meier survival analysis was performed to present the curves of total duration of labour by parity. After z-transformation of labour duration, multivariable linear regression was used to control for confounding and to identify independent associations between potential associated factors and the primary outcome of labour duration.

**Results:**

Overall, 1209 eligible women agreed to participate and were investigated. Rates of different intrapartum interventions were 27.4% in use of amniotomy, 37.9% in use of oxytocin, 53.0% in continuous electronic fetal monitoring, and 52.9% in epidural use, respectively. The curve of total duration of labour was significantly different between nulliparas and multiparas (*P* < .001). Of the 1209 participants, 983 (81.3%) women eventually achieved successful vaginal birth while 226 (18.7%) women ended in intrapartum caesarean section. The median duration of total stage of labour was significantly longer in the nulliparous group [9.38 (6.33,14.10) hours] than that in the multiparous group [5.08 (3.00,7.83) hours] (*P* < .001). The following factors were independently associated with longer duration of total stage of labour: epidural analgesia (*P* < .001), primiparity (*P* < .001), continuous electronic fetal monitoring (*P* = .035), and increased birth weight (*P* = .005).

**Conclusions:**

Intrapartum medical interventions become common obstetric practices in urban China. Multifactorial variables independently associated with longer duration of labour were identified, including epidural analgesia, primiparity, continuous electronic fetal monitoring, and increased birth weight. Further research is required to validate these variables and to determine the modifiable factors for labour management. And models of care with lower intervention rates such as midwife-led models of care should be developed and implemented in China.

## Background

Childbirth is a normal psychological process for the majority of women. The World Health Organization (WHO) defines normal labour as low risk throughout, spontaneous in onset, with the fetus in vertex position and ending with the mother and fetus in good condition following a spontaneous delivery [1]. Over the decades, however, there is an increasing global trend towards the widespread over-medicalisation of labour and childbirth in many parts of the world [2–4] . A range of labour practices have been applied to initiate, accelerate, terminate, regulate or monitor the physiological process of labour [5], thus leading to a technocratic model of care for normal childbirth with frequent use of interventions during labour and childbirth [6].

Miller et al. termed the excessive medicalisation as too much, too soon (TMTS), which has become a global threat to maternal, fetal, and newborn wellbeing [7]. For instance, rising rates of clinical interventions, such as caesarean section, routine cardiotocography, labour induction, augmentation, and routine episiotomy, have been shown to cause avoidable harm if overused [7–9]. Unnecessary childbirth interventions could be linked to negative consequences in maternal and child health and may cause substantial health-care costs [10, 11].

The causes of the rise in intrapartum interventions emerge as a complex multifactorial labyrinth involving health systems, care providers, women, societies, and even fashion and media [12–14]. Many common obstetric practices, however, are of limited or uncertain benefit for low-risk women in spontaneous labour [15]. Unnecessary routine interventions in labour are actually associated with further interventions and result in decreased rates of spontaneous vaginal birth [16, 17]. Bohren et al. reported that more than a third of women experienced mistreatment and were particularly vulnerable around the time of birth [18]. The increasing medicalisation of childbirth processes tends to undermine the woman’s own capability to give birth and negatively impacts her childbirth experience [5]. Satisfaction with women’s childbirth experience is related to personal expectations, support from caregivers, bonding with professionals, and women’s involvement in decision making [15, 19]. Hence, there is a growing call from the international community for considering reducing interventional approaches for intrapartum management of childbearing women in spontaneous labour [15, 17].

In China, the overall annual rate of caesarean deliveries reached 34.9% between 2008 and 2014 [20]. In some urban areas like Shanghai, the caesarean section rate declined from 67% in 2009 to 52% in 2014 [20], but still far beyond the recommended maximum level of 15% from the WHO [21]. The third phase of the WHO global survey reported that intrapartum caesarean section (ICS) rate in China accounted for 24.2% of the women who attempted trial of labour, much higher than that in other countries [22]. For example, it was reported that the ICS rate was 14.6% in India and 6.9% in Japan [22]. In Bernitz et al.’s study conducted in Norway, the ICS rate was 5.9% in the WHO partograph (control) group and 6.8% in Zhang’s guideline (intervention) group [23]. A national explorative study in Dutch reported the ICS variation was 13–15% for nulliparas and 5–6% for multiparas, respectively [11]. The above figures show that China is confronted with a more serious situation than other countries in terms of childbirth medicalisation. In addition, since the implementation of China’s two-child policy, numbers of women with advanced maternal age and other high risks have increased and posed great challenges on promoting normal labour and childbirth [24, 25].

In view of the substantial changes in women’s characteristics and medicalisation of labour management over the decades, Zhang et al. conducted a large contemporary cohort study in US and has established a new labour guideline since then [26]. Within this context, however, data on the characteristics of labour among Chinese women, their use of intrapartum interventions, and the association between women’s clinical characteristics and duration of labour are less well investigated. Therefore, the aim of this study was to investigate pregnant women’s characteristics, intrapartum interventions, duration of labour and its associated factors; and to compare the differences of these variables between nulliparas and multiparas in urban China.

## Methods

### Settings

A multi-center cross-sectional observational study was carried out in three tertiary university hospitals in urban China between 1 August 2018 and 31 January 2019. The three hospitals included Obstetrics and Gynecology Hospital, Pudong Hospital, and Huashan Hospital North, which were all affiliated to Fudan University. Maternity health care in these hospitals (also in whole China) share a similar obstetrician-led model. Chinese obstetricians provide antenatal, intrapartum, and postnatal care for pregnant women throughout their perinatal periods, while the midwives only work in labour and delivery units. Most of the midwives working there are nurses learning on the job. They need to follow obstetricians’ orders to deliver intrapartum care.

### Participants

The inclusion criteria of our study were as follows: (1) healthy women at term (37–41.6 gestational weeks); (2) singleton, vertex presentation; (3) with spontaneous onset of labour; and (4) with no maternal or fetal risk factors. The exclusion criteria of women recruitment were: (1) body mass index (BMI) ≥30 kg/m^2^ before pregnancy; (2) trial of labour after previous caesarean delivery; (3) presence of indications of caesarean section; and (4) with complications such as heart disease, hypertension, and gestational diabetes mellitus requiring control by medication.

Sample size was calculated using the sample size formula for a cross-sectional study [$n=\left({Z}_{\alpha /2}^2 pq\right)/{\delta}^2$] [27]. In this formula, (1) *n* represents the sample size; (2) *p* represents ICS rate; (3) *q* equals to (1-p); (4) *Z*_*α/2*_ equals to 1.96 with *α* valued as 0.05 and by a two-tailed test; and (5) *δ *represents an allowable error and equals to 0.1*p*. Considering 24.2% proportion of ICS in China reported in the WHO global survey [22], we required 1203 participants in the study as the final sample size.

**Figure 1:**
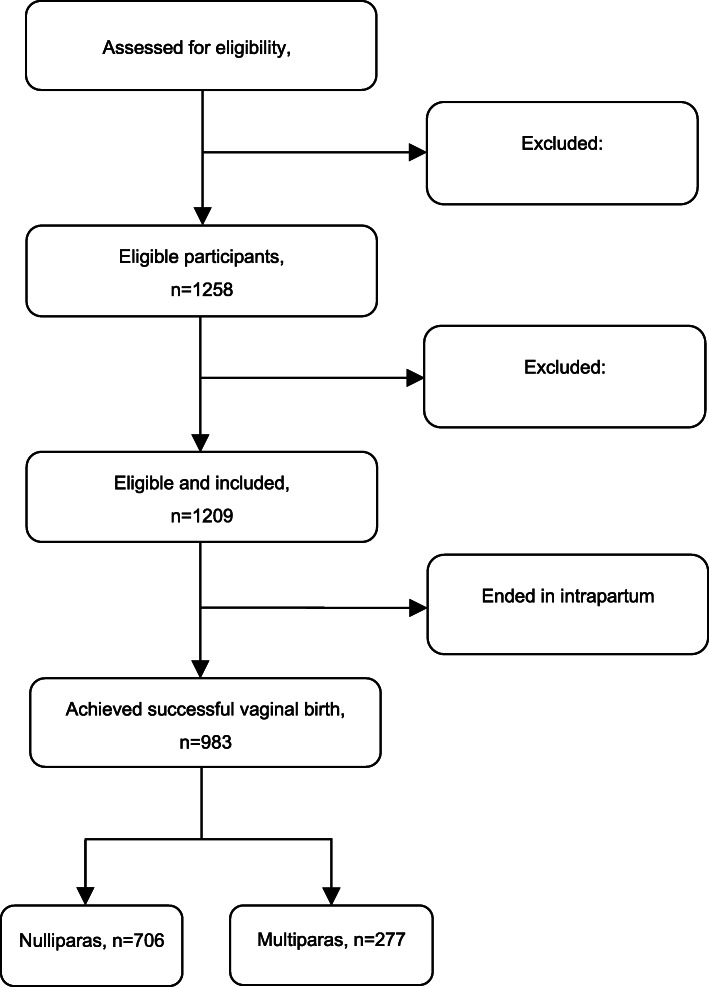
**Fig. 1** Flow chart of included and excluded participants

A total of 1523 women were approached and assessed for eligibility during the study period when they were admitted to the labour and delivery unit. Then 1258 women met the inclusion criteria and 49 women declined to participate. The rate of all eligible women was 82.6% (1258/1523). Finally 1209 women were recruited in our study (Fig. 1). There were no significant differences in basic characteristics between eligible women who agreed to participate and those who declined to participate (*P* > 0.05).

### Instrument

A tailored structured questionnaire (Additional file 1) was used to measure and describe women’s clinical characteristics, intrapartum interventions, and duration of labour. It was comprised of four parts including women’s baseline information, maternal conditions at admission of labour and delivery unit, labour progress and intrapartum interventions, and neonatal outcomes. The questionnaire was pre-tested in a similar population to the three study hospitals. Minor revisions of the questionnaire were made after the pretest.

### Data collection

Data were collected from women at the onset of labour. These women were followed until completion of delivering the baby. The onset of labour in this study was defined as regular, painful uterine contractions resulting in progressive cervical effacement and dilation [28, 29]. Labour was divided into three stages: first stage (including latent phase and active phase), second stage, and third stage. The sum of the three stages was equal to the total stage of labour.

The three research hospitals received a package of questionnaires along with invitation letters, consent forms and envelopes to ensure confidentiality of study participants. Ten midwives in labour and delivery units of the three hospitals were trained to be investigators of the study and filled in the questionnaires correctly. Data on maternal sociodemographic and clinical characteristics were obtained via chart review and inquiry from the consented women. Concurrently, four research nurse-midwives were recruited and trained to supervise and assist investigators, collect questionnaires, and check for consistencies and completeness of filled questionnaires. Envelopes with completed questionnaires were sent back directly to the research team. Questionnaires with any problems were returned to the investigators for re-survey.

The main outcomes for analysis were durations of labour including the first, second and total stages of labour. Other variables were women’s sociodemographic characteristics, use of intrapartum interventions, and birth outcomes.

### Data analysis

Body mass index (BMI) was calculated and classified as underweight (BMI < 18.5 kg/m^2^), normal weight (BMI between 18.5 and 24.9 kg/m^2^), overweight (BMI between 25.0 and 29.9 kg/m^2^), or obese (BMI ≥ 30.0 kg/m^2^) [30]. Participants were grouped into adequate gestational weight gain (GWG), inadequate GWG, and excessive GWG (Table 1).

**Table 1:** Category of gestational weight gain

**Underweight (BMI < 18.5 kg/m**^**2**^**)**		
IGWG^a^ (1)	AGWG^b^ (2)	EGWG^c^ (3)
< 12.7 kg	12.7 ~ 18.1 kg	> 18.1 kg
**Normal weight (BMI 18.5–24.9 kg/m**^**2**^**)**		
IGWG^a^ (4)	AGWG^b^ (5)	EGWG^c^ (6)
< 11.3 kg	11.3 ~ 15.9 kg	> 15.9 kg
**Overweight (BMI 25.0–29.9 kg/m**^**2**^**)**		
IGWG^a^ (7)	AGWG^b^ (8)	EGWG^c^ (9)
< 6.8 kg	6.8 ~ 11.3 kg	> 11.3 kg
**Obese (BMI ≥ 30.0 kg/m**^**2**^**)**		
IGWG^a^ (10)	AGWG^b^ (11)	EGWG^c^ (12)
< 5.0 kg	5.0 ~ 9.1 kg	> 9.1 kg

^a^*IGWG* Inadequate gestational weight gain (1)(4)(7)(10);

^b^*AGWG* Adequate gestational weight gain (2)(5)(8)(11);

^c^*EGWG* Excessive gestational weight gain (3)(6)(9)(12)

Descriptive statistics were calculated for sociodemographic and clinical characteristics of participants, which were summarized according to the following factors: age, educational level, household monthly income per capita, gestational weeks, prepregnancy weight category, gestational weight gain, intrapartum interventions, and labour duration. Continuous variables were analysed by means (SD) or by median (P_25_, P_75_). Categorical variables were analysed by number and frequency. For continuous variables, the statistical significance of the association between these factors and parities was tested using the t-test and Mann Whitney-U test. For categorical variables, the statistical significance of the association was tested using Chi-square test.

Kaplan-Meier survival analysis was performed to estimate the time-to-event outcome (vaginal birth). Log-rank test was used to compare the curves of total duration of labour between nulliparas and multiparas. Multivariable linear regression analysis was performed to explore the association between explanatory variables and the dependent variable (duration of labour), and to identify the relative importance of each independent variable to the outcome variable by controlling for the effects of other variables. Z-transformation was used to transform the non-normally distributed variable (duration of total stage of labour) into a normally distributed variable (N score of the duration of total stage of labour) using Blom’s Formula. The collected data were entered to Epidata Info and then exported to Statistical Package for Social Sciences (SPSS) Statistics Version 22.0. Statistical significance was set at a *p* value< 0.05.

## Results

A total of 1523 women admitted to the labour and delivery unit were approached and assessed for their eligibility and consent for the study, and 1209 participants provided informed consent. The sociodemographic characteristics of the study population are presented in Table 2. Of the 1209 consented participants, 841 (69.6%) women were nulliparous and 368 (30.4%) were multiparous women. The participants’ ages ranged from 20 to 48 years, with the mean maternal age of 29.86 (SD3.94) years. The majority of the participants were well educated, with 65.9% (*n* = 797) having completed a college or higher level degree. The mean gestational week at admission of this study was 39.56 (SD1.02) weeks, ranging from 37.0 to 41.5 weeks. The overall prevalence of underweight women was 18.9% (*n* = 228), whereas overweight and obesity accounted for 12.2% (*n* = 147).

**Table 2:** Characteristics of the participants by parity

Characteristics	Total (*N* = 1209)	Nullipara (*n* = 841)	Multipara (*n* = 368)	Statistic	*P* value
Age (years), n (%)				191.721^a^	<.001
20-24y	78 (6.5)	57 (6.8)	21 (5.7)		
25-29y	533 (44.1)	462 (54.9)	71 (19.3)		
30-34y	456 (37.7)	276 (32.8)	180 (48.9)		
35-39y	119 (9.8)	44 (5.2)	75 (20.4)		
≥ 40y	23 (1.9)	2 (0.2)	21 (5.7)		
Educational level, n (%)				9.067^a^	0.028
Junior high school or below	162 (13.4)	102 (12.1)	60 (16.3)		
Senior high/ technical school	250 (20.7)	169 (20.1)	81 (22.0)		
College graduate	597 (49.4)	438 (52.1)	159 (43.2)		
Postgraduate	200 (16.5)	132 (15.7)	68 (18.5)		
Household monthly income per capita, CNY, n (%)				0.605^a^	0.895
< 5000	95 (7.9)	69 (8.2)	26 (7.1)		
5000-7999	239 (19.8)	168 (20.0)	71 (19.3)		
8000-10,000	279 (23.1)	193 (22.9)	86 (23.4)		
> 10,000	596 (49.3)	411 (48.9)	185 (50.3)		
Gestational weeks, mean (SD)	39.56 (1.02)	39.66 (1.02)	39.34 (1.01)	5.159^b^	0.762
Prepregnancy weight category, n (%)				7.536^a^	0.057
Underweight (BMI < 18.5 kg/m^2^)	226 (18.7)	146 (17.4)	80 (21.7)		
Normal weight (BMI 18.5–24.9 kg/m^2^)	875 (72.4)	613 (72.9)	262 (71.2)		
Overweight (BMI 25.0–29.9 kg/m^2^)	100 (8.3)	78 (9.3)	22 (6.0)		
Obese (BMI ≥ 30.0 kg/m^2^)	8 (0.7)	4 (0.5)	4 (1.1)		
GWG, n (%)				6.665^a^	0.036
Inadequate GWG (1)(4)(7)(10)^c^	254 (21.0)	175 (20.8)	79 (21.5)		
Adequate GWG (2)(5)(8)(11)^c^	589 (48.7)	393 (46.7)	196 (53.3)		
Excessive GWG (3)(6)(9)(12)^c^	366 (30.3)	273 (32.5)	93 (25.3)		

*CNY* Chinese Yuan; *BMI* Body mass index; *GWG* Gestational weight gain

^a^ Chi-square test; ^b^*t* test;

^c^ Numbers in parentheses indicate number for category of gestational weight gain listed in Table 1

**Figure 2:**
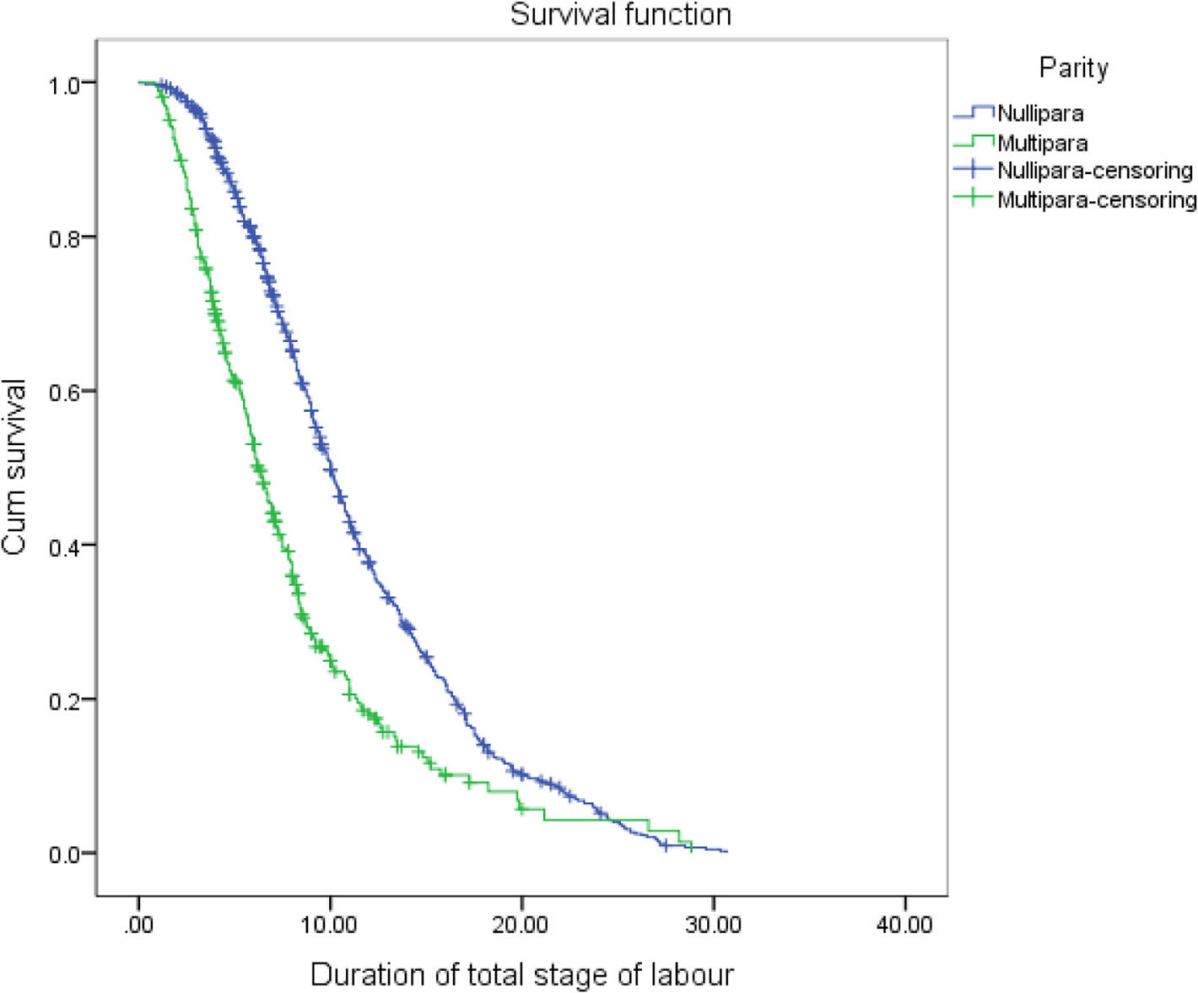
**Fig. 2** Duration of total stage of labour by parity (*n* = 1209)

Different intrapartum interventions were performed for the labouring women, including 27.4% (*n* = 331) of the amniotomy, 37.9% (*n* = 458) of use of oxytocin, 53.0% (*n* = 641) of the continuous electronic fetal monitoring (EFM), and 52.9% (*n* = 639) of use of epidural analgesia (Table 3). Of the 1209 participants, 983 women eventually achieved successful vaginal birth after trial of labour while 226 (18.7%) women ended in an ICS. These women undergoing ICS were censored during labour observation. Kaplan-Meier survival analysis unfolded that the duration of total stage of labour was significantly longer in nullliparous group than that in multiparous group (*χ*^2^=81.805, *P* < .001) (Fig. 2).

**Table 3:** Intrapartum interventions, birth outcomes and duration of labour

Variables	Total (*N* = 1209)	Nullipara (*n* = 841)	Multipara (*n* = 368)	Statistic	*P* value
Amniotomy, n (%)					
Yes	331 (27.4)	256 (30.4)	75 (20.4)	13.029^b^	<.001
No	878 (72.6)	585 (69.6)	293 (79.6)		
Use of oxytocin, n (%)					
Yes	458 (37.9)	391 (46.5)	67 (18.2)	87.036^b^	<.001
No	751 (62.1)	450 (53.5)	301 (81.8)		
Continuous EFM, n (%)					
Yes	641 (53.0)	522 (62.1)	119 (32.3)	90.847^b^	<.001
No	568 (47.0)	319 (37.9)	249 (67.7)		
Epidural analgesia, n (%)					
Yes	639 (52.9)	538 (64.0)	101 (27.4)	137.055^b^	<.001
No	570 (47.1)	303 (36.0)	267 (72.6)		
Mode of delivery, n (%)					
Vaginal birth	983 (81.3)	706 (83.9)	277 (75.3)	12.678^b^	<.001
ICS	226 (18.7)	135 (16.1)	91 (24.7)		
Neonatal birth weight, n (%)					
< 2500 g	193 (16.0)	150 (17.8)	43 (11.7)	2.308^b^	0.315
2500-4000 g	959 (79.3)	654 (77.8)	305 (82.9)		
> 4000 g	57 (4.7)	37 (4.4)	20 (5.4)		
Duration of 1st SOL (706/277)^a^, median (P_25_, P_75_)	7.00 (4.00, 11.00)	8.50 (5.50, 13.00)	4.50 (2.58 7.00)	50,391.000^c^	<.001
Duration of 2nd SOL (706/277)^a^, median (P_25_, P_75_)	0.63 (0.32, 1.07)	0.78 (0.45, 1.18)	0.28 (0.16, 0.53)	42,825.000^c^	<.001
Duration of total SOL (706/277)^a^, median (P_25_, P_75_)	8.00 (4.83, 12.25)	9.38 (6.33, 14.10)	5.08 (3.00, 7.83)	46,859.500^c^	<.001

*EFM* Electronic fetal monitoring; *ICS* Intrapartum cesarean section; *SOL* Stage of labour

^a^ Numbers in parentheses indicate number for whom this information was available (nullipara/multipara); ^b^ Chi-square test; ^c^ Mann-Whitney U test

Of the 983 participating women who delivered vaginally, 706 women were nulliparous and 277 women were multiparous. The median duration of first stage of labour was 8.50 (5.50, 13.00) hours in the nulliparous group, and 4.50 (2.58, 7.00) hours in the multiparous group, making significant differences between the two groups (*P* < 0.001). The median duration of second stage of labour in the nulliparous group [0.78 (0.45, 1.18) hours] was significantly longer than that in the multiparous group [0.28 (0.16, 0.53) hour](*P* < 0.001). The median duration of total stage of labour was 9.38 (6.33, 14.10) hours in the nulliparous group, and 5.08 (3.00, 7.83) hours in the multiparous group, giving significant differences between the two groups (*P* < 0.001). We found no significant differences in the neonatal birth weight between the two groups (*P* > 0.05) (Table 3).

Compared with nulliparous women, multiparous women in this study were less likely to have an amniotomy (75 [20.4%] versus 256 [30.4%]; *P* < .001), less likely to be administered oxytocin (67 [18.2%] versus 391 [46.5%]; *P* < .001), less likely to receive continuous EFM (119 [32.3%] versus 522 [62.1%]; *P* < .001), and less likely to use epidural analgesia (101 [27.4%] versus 538 [64.0%]; *P* < .001) (Table 3).

Of the 983 women who eventually achieved successful vaginal birth after trial of labour, we analysed the associations between potential predictors and the outcome variable of labour duration. Due to skewness of the distribution of labour duration, data was z*-*transformed into normal distribution before analysed using multivariable linear regression. The potential associated factors were then entered into the multivariable linear regression model. In the final model, the following factors were independently associated with longer duration of total stage of labour: epidural analgesia (*P* < .001), primiparity (*P* < .001), continuous EFM (*P* = .035), and increased birth weight (*P* = .005) (Table 4).

**Table 4:** Multivariable linear regression of factors associated with duration of total stage of labour

Variables	β^a^	95%CI	*t *Test	*P* value
Age	0.01	(−0.01, 0.02)	0.98	0.328
Epidural analgesia^b^	0.85	(0.73, 0.96)	15.63	< 0.001
Parity^c^	−0.54	(−0.62, -0.37)	−7.84	< 0.001
Continuous EFM^b^	0.12	(0.02, 0.23)	2.11	0.035
Amniotomy^b^	0.09	(−0.03, 0.21)	1.49	0.136
Birth weight	0.20	(0.06, 0.34)	2.81	0.005

*EFM* Electronic fetal monitoring; *CI* Confidence interval;

^a^Due to skewness of the distribution of labour duration, data was z-transformed before analysed using multivariable linear regression; ^b^Binary variable (yes = 1, no = 0); ^c^Binary variable (nullipara = 0, multipara = 1)

## Discussion

This was a multi-center cross-sectional study conducted in three tertiary university hospitals in Shanghai, China. We found that intrapartum medical interventions become common obstetric practices in urban China. Also multifactorial variables associated with duration of labour were identified in our study.

It was found in this study that the intrapartum interventions accounted for 27.4% in amniotomy, 37.9% in oxytocin administration, and 53.0% in the use of continuous EFM (Table 3), unfolding that these procedures are common interventional practices in China’s obstetric settings. Meanwhile, we found in our study that the overall epidural rate was 52.9%, whereas an Australian study reported that the overall rate of epidural analgesia was 35.9% [31]. Beyond that, our study showed that the rate of ICS was 18.7%. This rate is lower than Lumbiganon et al.’s report of 24.2% in China [22], but still much higher than Bernitz et al.’s report of 5.9–6.8% in Norway [23]. Our findings illustrated a technocratic model of care for normal childbirth in China, which could be explained by the fact that China’s maternity care is an facility based obstetrician-led model [32–34]. The regulations and organisational framework of maternity services constitute contextual factors fostering medical interventions during labour [35].

We found in our study that compared with nulliparas, multiparas were less likely to receive intrapartum interventions including amniotomy, oxytocin, continuous EFM, and epidural analgesia (Table 3). Similar findings have been previously reported by Grylka-Baeschlin et al.’s study, where there were lower intervention rates in multiparas than in primiparas [36]. For neonatal birth weight, we found no statistically significant differences between the nulliparas group and the multiparas group, which indicated that parity did not affect this variable significantly. Admittedly, further studies still need to be conducted on account of the causality challenge of this study. Meanwhile, our study also identified the independent multifactorial variables associated with labour duration, ranging from epidural analgesia, parity, continuous EFM, and birth weight (Table 4) .

Firstly, by adjusting for confounding, we found an association between labour duration and epidural analgesia. Our study indicated that use of epidural was associated with longer duration of labour. This finding was in concordance with Turner et al.’s findings [31]. Although epidurals may reduce pain during labour more effectively than any other form of pain relief, it may also be associated with unwanted effects like prolonged labour, hypotension, drowsiness and fever [37]. Therefore, to ensure a positive childbirth experience, women should be allowed to make decisions about their pain management. Epidural analgesia can be one of the options to choose when required by labouring women [5]. Beyond that, nonpharmacologic methods such as some relaxation therapies and continuous labour support should also be offered to women, in order to help them feel more in control and satisfied with their labours [38].

Secondly, the results of our study demonstrated that labour duration was significantly longer in nulliparous women than that in multiparous women, which was consistent with Chen et al.’s finding [39]. Concurrently, durations of the first, second, and total stage of labour in our study was similar to those reported by Chen et al. [39]. Then we found that parity was an independent variable associated with labour duration.

In addition, we found an association between continuous EFM and duration of labour. On the one hand, continuous EFM might restrict women from moving freely during labour and could be associated with prolonged labour. Previous studies demonstrated that freedom to move and adopt upright positions in labour results in a range of physical and psychological benefits for women, including reduced risk of caesarean section, increased sense of control during labour and increased satisfaction with the birth experience [40]. Therefore, women should be encouraged to be mobile and to adopt comfortable positions of their choice so as to gain a positive childbirth experience. On the other hand, continuous EFM might be performed for labouring women because of prolonged labour. Therefore, temporal direction of the association between continuous EFM and duration of labour could not be clearly ascertained.

Furthermore, we found in our study that increased birth weight was an independent factor associated with longer duration of labour, which is consistent with Leftwich et al.’s study conducted in the US [41]. Based on this research evidence, it is reasonable to allow longer time for labour progression when a larger fetus is suspected [41]. Beyond that, this finding also highlighted the importance to design and implement weight management interventions that may prompt women to conceive an appropriate size of fetus and to experience a normal labour progression.

However, it is notable that we did not find any association between duration of labour and amniotomy (Table 4). It can be assumed that amniotomy might be performed for women who had presented a prolonged labour but labour was not prolonged due to amniotomy. Interestingly, however, Vadivelu et al. have shown that amniotomy was associated with a shorter labour duration compared with conservative management in women with singleton pregnancies [42]. Conversely, Chen et al. found that in nulliparas, the average time of first stage of labour and total labour duration increased due to amniotomy [39]. The association between amniotomy and labour duration varied due to different research designs and inter-study heterogeneity. Yet Chen et al.’s study illustrated that women with medical interventions were more likely to have prolonged labour processes [39]. And there is no evidence that these interventions can improve childbirth experience for women who have had a prolonged labour [43]. Therefore, amniotomy, as one of the most common interventions in modern obstetric practice, should be performed with caution instead of being used routinely during labour management.

Meanwhile, we found in our study that maternal age was not associated with duration of labour (Table 4). However, this does not mean that the variable of age is unrelated to labour duration and management of labour. Greenberg et al. stated that older women had a persistently higher likelihood of experiencing longer labour and prolonged labour than younger women [44]. Given these contradictory findings, further research is needed to clarify the association between maternal age and duration of labour.

From all discussed above, we can see in this study that various medical interventions prevail in Chinese urban obstetric settings. In concordance with our findings, previous studies have also noted that there is a continually increasing trend in routine use of medico-technical interventions [17, 45, 46]. Lu et al. also pointed out the current situation of frequently used intrapartum interventions which may substantially distort the labour pattern [47]. According to International Confederation of Midwives (ICM), midwives are the most appropriate care providers to attend childbearing women [48]. One Cochrane Review by Sandall et al. suggests that women who received midwife-led continuity models of care were less likely to experience intervention and more likely to be satisfied with their care than women who received other models of care [49]. As such, models of care with lower intervention rates such as midwife-led models of care should be developed and implemented in order to rectify the medicalisation of childbirth in China.

### Limitations

One of the limitations of our study is the causality difficulty of ascertaining the association between duration of labour and the several explanatory variables due to the cross-sectional nature of our collected data. Meanwhile, we did not address all the possible predictive factors that could affect women’s labour duration, though we had attempted to control some of the identifiable confounders. Another limitation is that our study only included women with vertex presentation, term birth, and spontaneous onset of labour, thus limiting its generalizability and applicability to other women who were excluded. In this study, women’s cervical dilatation was assessed by digital vaginal examination, which was subjective and might cause some unavoidable measurement errors. In spite of these limitations, the strengths of our study include the fact that women’s clinical data were collected through medical charts prospectively, from women’s onset of labour till completion of delivery. As such, the information bias is considered to be minimal.

## Conclusion

Currently in urban China, intrapartum interventions for childbearing women mainly consist of various medico-technical measures during labour. Among them, continuous EFM, amniotomy, and oxytocin treatment have become common practices. Also, the magnitude of epidural analgesia in our study represented a higher proportion among labouring women, indicating that women in urban China are on the one hand having more choices of and access to labour analgesia services, and on the other hand are receiving more pharmacologic methods for labour pain management. Meanwhile, epidural analgesia, primiparity, continuous EFM, and increased birth weight are associated with longer duration of labour. Further research is required to validate these variables and to determine the modifiable factors for labour management. In order to limit unnecessary intrapartum medical interventions and to improve womens’ childbirth experience, obstetric care providers should take full account of these factors during prenatal counseling and in the process of labour management for childbearing women. Furthermore, there is an urgent need for developing models of care with lower intervention rates such as midwife-led models of care to rectify the medicalisation of childbirth in China.

## Supplementary information

**Additional file 1.** Questionnaire.

## Data Availability

The datasets used and/or analyzed during the current study are available from the corresponding author on reasonable request.

## Abbreviations

BMI: Body mass index
CI: Confidence interval
CNY: Chinese yuan
EFM: Electronic fetal monitoring
GWG: Gestational weight gain
ICM: International confederation of midwives
ICS: Intrapartum caesarean section
SOL: Stage of labour
WHO: World health organization
