# Correction to: Pregnant women’s clinical characteristics, intrapartum interventions, and duration of labour in urban China: a multi-center cross-sectional study

**DOI:** 10.1186/s12884-020-03104-6

**Published:** 2020-07-27

**Authors:** Chunyi Gu, Xiaojiao Wang, Zhijie Zhang, Simone Schwank, Chunxiang Zhu, Zheng Zhang, Xu Qian

**Affiliations:** 1 Department of Maternal, Child and Adolescent Health, School of Public Health, Fudan University, Shanghai, China; 2 Department of Nursing, Obstetrics and Gynaecology Hospital of Fudan University, Shanghai, China; 3 Department of Epidemiology, School of Public Health, Fudan University, Shanghai, China; 4 Department of Women and Children’s Health, Reproductive Health, Karolinska Institutet, Stockholm, Sweden; 5 Global Health Institute, Fudan University, Shanghai, China

**Correction to: BMC Pregnancy Childbirth (2020) 20:386**

**https://doi.org/10.1186/s12884-020-03072-x**

Following publication of the original article [1], the authors identified an error in Fig. 1. The correct figure is given below.

**Fig. 1:**
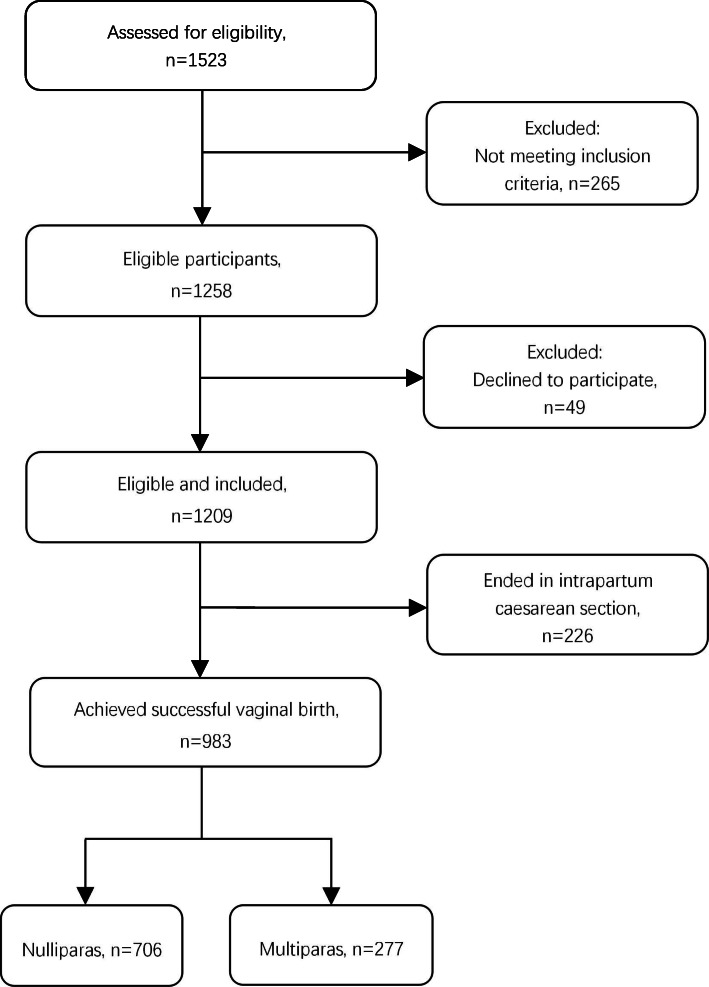
Flow chart of included and excluded participants

The original article has been corrected.
