# Supplementary material for: Pregnant women’s clinical characteristics, intrapartum interventions, and duration of labour in urban China: a multi-center cross-sectional study
Source: BMC Pregnancy Childbirth. 2020 Jul 2;20:386. doi: 10.1186/s12884-020-03072-x (PMC7330978; doi:10.1186/s12884-020-03072-x)
Supplement: Supplementary file 1 — Additional file 1. Questionnaire. [file 12884_2020_3072_MOESM1_ESM.docx]

问卷编号(NO.): 日期(DATE):

**孕产妇特征、产时干预、产程时限和分娩结局的调查问卷**

**Questionnaire survey on women’s characteristics, intrapartum intervention, duration of labour, and childbirth outcomes**

1. **孕产妇一般情况 Women’s baseline information**
2. 年龄： 岁 Maternal Age:

2. 文化程度（ ） A. 中专 B. 大专 C. 本科 D. 硕士及以上 E. 其他

Education (last grade completed):

1. Junior high school or below B.senior high/technical school C. college graduate D. postgraduate

3. 家庭人均月收入（ ）A.3000元以下 B.3000～5000元 C.5000～8000元 D.8000～10000元 E.10000元以上

Monthly income：

1. under 3000 yuan B.3000-5000 yuan C.5000-8000 yuan D.8000-10000 yuan E.above 10000 yuan
2. 分娩孕周：______周 孕次___产次__ Gestational week： Gravida____Para____

5. 孕前体重：________kg 入室时体重：_______kg 身高：_______m

Pre-pregnancy weight (kg) :

Maternal weight at admission of labour and delivery unit (kg) :

Maternal height (m) :

1. **入室情况 Maternal conditions at admission of labour and delivery unit**

1. 入室时间：_____年_____月_____日_____时_____分

Date of admission: _____/_____/_____ Time :_____:_____

2. 入室指征（可多选）：（ ）

A. 见红 B.不规律宫缩： 间隔 持续 C. 其他：

Indications for admission:

1. Vaginal bleeding (show)
2. Regular contractions: interval______duration_______

C.other ( please specify):_________

3. 入室时宫口扩张情况：宫口扩张______cm 胎先露下降______cm

Dilatation of cervix (cm):________ Station:________

1. **产程进展及干预 Labour progress and intrapartum interventions**
2. 临产情况 Labor information

1. 临产时间：_____年_____月_____日_____时_____分

Time of onset of labor:_____(Year)/_____(Month)/_____(Day) Time:_____:_____

2. 破膜情况（ ）

A. 胎膜早破

B. 胎膜自破

C. 人工破膜， 指征：□综合引产 □加速产程 □了解羊水性状

Rupture of Membranes :

A. Premature rupture of membranes (PROM)

B. Spontaneous rupture of membrane (SROM)

C. Artificial rupture of membranes (AROM)

1. 破膜时间：_____年_____月_____日_____时_____分（共_____时_____分）

Time of membrane rupture _____(Year)/_____(Month)/_____(Day) Time:_____:_____（ ____h_____min in total ）

1. 宫口扩张进展、胎先露及时间 Cervical Examination

| 时间（24h制）  Time (24h) | 宫口扩张（cm）  Dilatation (cm) | 先露下降程度（cm）  Station |
| --- | --- | --- |
| _______时_______分  Time:______:_______ |  |  |
| _______时_______分  Time:______:_______ |  |  |
| _______时_______分  Time:______:_______ |  |  |
| _______时_______分  Time:______:_______ |  |  |
| _______时_______分  Time:______:_______ |  |  |
| _______时_______分  Time:______:_______ |  |  |
| _______时_______分  Time:______:_______ |  |  |
| _______时_______分  Time:______:_______ |  |  |
| _______时_______分  Time:______:_______ |  |  |
| _______时_______分  Time:______:_______ |  |  |

1. **产程干预 Interventions during labor**
2. 是否应用产程图记录产程进展（ ） A.是 B.否

Use of partogram ( ) A.yes B.no

2. 在产程进展过程中进行了（可多选）：（ ）

A. 人工破膜 B. 静滴催产素 C. 产钳助产 D.徒手转胎位

E. 会阴切开 □未切开， 度撕裂 □会阴侧切 □会阴正中切

F. 其他

What measures were taken in delivery? (select all that apply)

A. AROM B.oxytocin infusion C. forceps delivery D.manual rotation

E. episiotomy □laceration, degree:_______□mediolateral □median

F. others

3. 产程管理中胎心监测采用（可多选）：（ ）

A.整个产程持续进行胎心监护

B.第一产程特殊治疗下（如催产素滴注）持续胎监

C.第一产程无特殊情况下间断多普勒胎心听诊

D.第二产程持续胎心监护

E.第二产程间断胎心监护

Fetal monitoring during labour:

A.continuous electronic fetal monitoring during 1st stage of labor

B.intermittent auscultation fetal monitoring with doppler in the 1^st^ stage of labor

C.continuous electronic fetal monitoring in the 2^nd^ stage of labor

D. Intermittent fetal monitoring in the 2^nd^ stage of labor

4. 分娩方式（ ）

A. 阴道分娩

B. 阴道试产转为剖宫产，剖宫产指征:

Mode of delivery:

A.Vaginal birth

B. Intrapartum cesarean section, indications:

5. 分娩时间：_____年_____月_____日_____时_____分

Date of birth: _____/_____/_____ Time ____:_____

6. 产程情况 Labour condition

第一产程开始时间：_____时_____分；Start of 1^st^ stage:____:_____

第二产程开始时间：_____时_____分；Start of 2^nd^ stage: ____:_____

第三产程开始时间：_____时_____分；结束时间：_____时_____分

Start of 3^rd^ stage: ____:_____ End of 3^rd^ stage: ____:_____

总产程时间： 时 分 Total length of labor:_______h_____min

1. 新生儿情况 Neonatal outcomes
2. 新生儿性别（ ） A. 男 B. 女 Newborn gender: A. male B. female
3. 出生体重：_________g Birth weight (g) : ________

1. 新生儿Apgar评分：1分钟________分；5分钟_________分

Neonatal Apgar score:____at 1 min;____at 5 min
